# Cross-sectional investigation of gender differences in health-related quality of life among HIV patients: implications for gender mainstreaming in HIV management

**DOI:** 10.11604/pamj.2021.39.201.24420

**Published:** 2021-07-15

**Authors:** Daprim Samuel Ogaji, Obiageli Philomena Igwebuike

**Affiliations:** 1 Department of Preventive and Social Medicine, University of Port Harcourt, Choba, Nigeria,; 2 Africa Centre of Excellence in Public Health and Toxicological Research (ACE-PUTOR), University of Port Harcourt, Choba, Nigeria

**Keywords:** HIV, health-related quality of life, HRQoL, WHOQoL HIV–BREF, gender, Nigeria

## Abstract

**Introduction:**

health-related quality of life (HRQoL) can be affected by the existence of long-term medical conditions. This study compared the HRQoL of male and female patients living with the human immunodeficiency virus (HIV) who sought care at the antiretroviral clinic in a tertiary hospital.

**Methods:**

a comparative cross-sectional study with 512 female and 512 male HIV outpatients receiving care at the antiretroviral clinic in the University of Port Harcourt Teaching Hospital. The WHOQoL-HIV-BREF which conceptualized HRQoL as a function of six factors - physical, psychological, social, environment, independence and spiritual health was administered. The gender difference in HRQOL was determined by the independent sample t-test, mean difference and standardized mean difference in items and domain scores. Meta-analytic approach was used to deduce the overall potential effect of gender on HIV infection. Multivariate linear regression analyses were used to control for potential confounders of HRQoL among the study participants.

**Results:**

the mean age of the sampled population was 35.9 ± 11.8 years for the male and 35.3 ± 9.8 years for the female category. Male HIV patients reported significantly higher mean HRQoL scores across all domains of the scale except the spiritual domain. The 4.51% (95% CI of 3.63 to 5.39%) overall difference was statistically significant (p<0.001). Other factors associated with good HRQoL were marriage status, monogamous family type and a higher level of education.

**Conclusion:**

the significantly lower HRQoL among female HIV patients calls for a multiprong approach towards strengthening gender mainstreaming in the management and control of HIV patients in Nigeria.

## Introduction

Human immunodeficiency virus (HIV) infections remain a significant contribution to morbidity, disability and mortality worldwide. Globally, mortality from the human immunodeficiency virus (HIV) reached its peak of 1.95 million deaths in 2006 while the annual incidence of new infections peaked earlier in 1999 at 3.16 million [1]. The high prevalence of HIV as well as the increasing longevity experienced by people living with HIV/AIDS (PLWHA) are linked to increasing access to anti-retroviral therapy (ART) and decline in deaths attributable to HIV/AIDS [1].

Global trends reveal decreasing HIV mortality and incidence of new cases from 2006 to 2017 while prevalence and ART coverage have increased over the period [2]. By 2017, there was a 48.5% higher new HIV infection and 3.7% HIV deaths among females living in sub-Saharan Africa [1]. While the preponderance of global HIV cases and deaths from acquired immune deficiency syndrome (AIDS)-related causes occur in sub-Saharan Africa, 3.3% of global disability-adjusted life years was attributable to HIV/AIDS [3]. Nigeria which ranked third position behind South Africa and India, was reported to have contributed 9,012 of the global disability-adjusted life years (DALYs) and 11.1% of global HIV/AIDS deaths [3].

Nigeria maintains a large pool of PLWHA despite the decreasing incidence of new infections over the last few decades. The trend in prevalence shows an increased HIV prevalence in Nigeria from 1.8% in 1991 until it attained a peak national prevalence of 5.8% in 2001 and then gradually declined to a prevalence of 1.4% among adults aged 15 - 49 years in 2018 [4]. The distribution of cases across geopolitical zones in Nigeria from the 2018 Nigeria HIV/AIDS Indicator and Impact Survey (NAIIS) identified the south-south zone as having the highest prevalence of 3.1% among adults aged 15 - 49 years. Rivers State which is one of the six states in this zone reported the highest proportion of PLWHA in the recent seroprevalence survey with a prevalence of 3.4% [4].

Quality of life (QoL) represents how happy or bothered people feel to various aspects of their life. This construct reflects how personal perceptions of goals, expectations, standards and concerns relate to the culture and value system of the environment [5]. This construct is influenced by the physical health, psychological state, level of independence, social relations, and one´s relationship with the essential elements of the environment. The multi-dimensional health-related quality of life (HRQoL) demonstrates the impact of an individual´s health status on the QoL [5,6]. Being a critical outcome measure among PLWHA, HRQOL demonstrates the impact of HIV disease and its management on the physical, social, and psychological wellbeing of PLWHA [6]. Valid measurement of HRQoL therefore integrates the social and biomedical factors that influence human health and wellbeing [5]. The increasing life expectancy of PLWHA makes it imperative to measure and monitor their HRQoL and identify its determinants. Some patients are affected by the symptoms of HIV, the emergence of opportunistic infections or the side effects of antiretroviral therapy [3,4,7]. While improved access to highly active antiretroviral therapy (HAART) had resulted in significant improvement in the clinical and laboratory outcomes in HIV/AIDS patients; the goal of treatment transcends survival to improving the quality of life of PLWHA.

The HRQoL of HIV patients can be influenced by disease-related factors (CD4+ count, viral burden, HIV disease stage); psychosocial factors (social support, coping and disclosure); socio-demographic characteristics (age, gender, employment and level of education) [7]. Understanding the magnitude and how these factors affect the HRQoL will enable health planners, managers, and policymakers design interventions for improving the HRQoL of PLWHA. The feminization of the HIV pandemic has strong evidence in the local setting as women are increasingly more vulnerable to HIV infection and its consequences. In Nigeria, there were disproportionately higher numbers of new HIV infections (128,000 vs. 88,800) and deaths (87,500 vs. 81,600) from AIDS in 2017 among women [1]. This trend is accentuated by biological, social, cultural, religious, economic, and legal factors [8]. This is despite the salutary observation that female HIV patients are twice as likely to achieve viral load suppression as males [4].

Gender differences in HRQoL among PLWHA are not well documented in Nigeria and especially in the study area which bears the brunt of the HIV burden in Nigeria [4]. Establishing gender disparity in HRQoL will support the policy dialogue for gender mainstreaming in the provision of HIV care services as a strategy for improving the lives of PLWHA. This study measured and compared the HRQoL of male and female HIV/AIDS patients receiving care in a referral hospital. It hypothesized that the physical, psychological, level of independence, social relationship, environmental health and spiritual wellbeing of male and female HIV patients on treatment are identical.

## Methods

**Research design:** the study is a comparative, cross-sectional study.

**Study setting:** this study was conducted in the anti-retroviral (ARV) clinic of the University of Port Harcourt Teaching Hospital (UPTH). The ARV clinic in UPTH is run by a multidisciplinary health team from the departments of internal medicine, haematology, community medicine, counselling, and social welfare departments. Each clinic session starts with general health education sessions conducted by the nurses and counsellors after which the clients either see a doctor or simply proceed to the pharmacy for drug refills. New clients undertake comprehensive review and investigation before those that meet set eligibility criteria are commenced on HAART. This ARV clinic attends to about 90 patients daily, runs four times in a week and accommodates the state´s largest HIV database of over 9,000 patients of which 4,767 of them are active on ART.

**Study population:** the study population were adult, ambulatory HIV patients, who have been consistent with their care at the ARV clinic in UPTH for more than 6 months and gave consent to participate in the study. Pregnant women and severely ill/debilitated patients were excluded.

**Sample methodology:** the minimum sample size was determined for a two-tail parallel design based on a hypothesis of equality of both groups in the measured numeric outcome. The required sample size was calculated as [9]:

n per group=2(Zα2+Zβ)2*SD2(μ1−μ2)2

Where: n = sample size in each group (assumed equal sized groups); Z_α/2_= standard normal deviate corresponding to α level of 0.025 in each tail = 1.96; Z_β_= 0.84 for a power of 80%; μ1 and μ2 are the means of the respective groups; SD is the pooled standard deviation of both groups estimated as:

$$\sqrt{\frac{{\left(S_{1}+S_{2}\right)}^{2}}{2}}$$

A previous study conducted in East Africa reported a mean HRQoL score of 81.2 ± 14.2 for males and 77.1 ± 17.4 for females [10].

$$n_{2}=n_{2}=\frac{2x{\left(1.96+0.84\right)}^{2}x499.28}{{\left(81.2-77.1\right)}^{2}}\approx 465$$

Increasing this by 10% in view of potential nonresponse or inappropriately completed questionnaires produced a minimum sample size of =512. The participants were recruited by stratified random sampling from the population of those receiving ARV in the centre.

**Study variables:** the independent variables in this study were gender, age, marital status, educational status, HIV clinical status, employment status, perceived health status, family type while the dependent variables were scores along the item domains and entire WHOQoL-HIV-BREF scale.

**Data collection:** the data was collected over an 8-week period (July 8^th^ to August 30^th^, 2019) and about 17 clients were recruited for each group per day from the record department´s list of those given appointment on each clinic day. The questions were read out for those who could not read and interpretation in vernacular were done when necessary. Those who are educated completed their questionnaires and sought assistance, when necessary, from members of the research team. The study instrument was the WHOQoL-HIV-BREF questionnaire [11] which conceptualized HRQoL as a function of six factors - physical health (4 items), psychological well-being (5 items), level of independence (4 items), social relationship (4 items), environmental health (8 items), and spiritual health (4 items). Each item has a five-point Likert response scale where 1 indicates low perception score of HRQoL and 5 a high perception score.

**Data analysis:** the data was entered into SPSS version 21.0 [12] where it was cleaned, responses of negatively worded questions (Q3, Q4, Q5, Q8, Q9, Q10 and Q31) were reversed, scores transformed to percentages (0 to 100 scale) with higher scores indicating better health, missing values substituted with series mean before analysis. Item scores were summed to compute for the domains and entire scale scores, each ranging from 0 to 100. The internal consistency reliability of the questionnaire was assessed by the Cronbach´s alpha coefficient for the entire scale. The face and content validities were assured during the pilot phase of the research while the construct validity was determined by the item response characteristics, item-total, and domain-total partial correlation.

Exploratory data analyses involved the calculation of frequencies and percentages for categorical variables, means and standard deviations for continuous variables and the presentation of a forest plot. The gender differences across the items, domains and entire WHOQoL-HIV-BREF scale was computed by the difference and standardized difference between both means. Meta-analysis conducted with the WINPEPI statistical software [13] and explored the statistical effect of gender on HRQoL scores along with items and the entire WHOQoL-HIV-BREF scale. The independent t-test was used to test the significance of the difference in the mean scores of males and females while other factors that may be associated with HRQoL were controlled for in the multivariate linear regression analysis with the significant level set at p<0.05.

**Ethical clearance/permission/consent:** ethical approval with reference UPH/CEREMAD/ REC/MM61/048, dated 23^rd^ May 2019 was obtained from the Ethical and Research Committee of the University of Port Harcourt. As these involved human subjects, a subsequent ethics approval with reference - UPTH/ADM/90/511/VOL-X/845 and dated 2^nd^ October 2019 was obtained from the ethics unit of the University of Port Harcourt Teaching Hospital. Permission was also sought and obtained from the department of internal medicine and a written informed consent was also obtained from each participant before each interview. All study participants were informed of the benefits of the study and assured of their confidentiality.

## Results

A total of 1,024 of the 1,034 questionnaires distributed were returned, giving a questionnaire response rate of 99%. Those that were not returned were from the self-administered group who left the clinic without returning the completed questionnaires. The mean item nonresponse rate was 3.9% with a range of 2.1% - 11.2% across all items in the scale. The Cronbach´s alpha coefficient for the entire scale was 0.92, and for the domains ranged from 0.35 (independence) to 0.78 (psychological). The item-total correlation ranged from 0.2 (Q20) to 0.7 (Q24) while that of the domain-total ranged from 0.5 (level of independence) to 0.8 (psychological). The inter-domain correlation ranged from 0.3 (between environment and spiritual) to 0.7 (environment and social relationship).

From Table 1, more of the participants in this study were single (47.1%), had attained secondary education (53.8%) unemployed (69.9%). The majority of the respondents were clinically asymptomatic (62.0%) and rated their current health status as good to excellent (90.3%). Table 2 shows the level and comparison of the mean scores of the various attributes which define HQROL for male and female HIV patient. The least rated item on the scale was related to financial sufficiency for every daily need (male = 48.7% and female = 43.7%) while the highest rated item for both groups was extent patient was not bordered by public reaction to their HIV status (male = 79.2% and female = 79.0%). Gender-related statistically significant differences were observed in 23 of the 31 items in the WHOQoL-HIV-BREF scale, and these were all in favour of the male. The most prominent gender difference was in the capacity for working, which recorded a mean difference of 10% (95% CI: 3.0, 17.0). In the other 8 items in the scale, there were observed gender-based differences, but these were not statistically significant. Out of these 8 items, male fared better in 6, female in 1 (satisfaction with health services) and both groups had the same score in 1 (assurance of living).

**Table 1:** patients' background characteristics

Variable	Male - frequency (%)	Female - frequency (%)	Total frequency (%)
**Age**			
≤ 40 years	357 (69.7)	391 (76.4)	748 (73.0)
> 40 years	155 (30.3)	121 (23.6)	276 (27.0)
**Marital status**			
Single	268 (52.3)	214 (41.8)	482 (47.1)
Married	209 (40.8)	254 (49.6)	463 (45.2)
Separated/divorced	12 (2.3%)	13 (2.5)	25 (2.4)
Widowed	23 (4.5)	31 (6.1)	54 (5.3)
**Level of education**			
No formal education	9 (1.8)	16 (3.1)	25 (2.4)
Primary	55 (10.7)	69 (13.5)	124 (12.1)
Secondary	261 (51.0)	290 (55.6)	551 (53.8)
Tertiary	187 (36.5)	137 (26.8)	324 (31.6)
**Family type**			
Monogamous	389 (76.0)	388 (75.8)	777 (75.9)
Polygamous	123 (24.0)	124 (247)	247 (24.1)
**Employment status**			
Yes	381 (74.4)	335 (65.4)	308 (30.1)
No	131 (25.6)	177 (34.6)	716 (69.9)
**Perceived heath status**			
Not good	33 (6.4)	62 (12.1)	95 (9.3)
At least good	479 (93.6)	450 (87.9)	929 (90.3)
**Clinical status**			
Asymptomatic	310 (60.5)	325 (63.5)	635 (62.0)
Symptomatic	202 (39.5)	187 (36.5)	389 (38.0)

**Table 2:** mean HRQoL item scores in male and female HIV patients

Item	HRQoL criteria	Scores - mean (SD)		MD (95% CI)	t-test (df=1022)	p-value
		Male (n=512)	Female (n=512)			
1	Perceived QoL	73.6 (14.1)	68.6 (20.5	5.0 (2.8, 7.2)	4.55	>0.001
2	Perceived health	70.5 (16.6)	63.5 (23.7)	7.0 (4.5, 9.5)	5.47	>0.001
3	No restriction	77.3 (24.5)	75.8 (27.8)	1.4 (-1.8, 4.7)	0.88	0.378
4	No worries	76.6 (25.1)	70.7 (28.5)	5.9 (2.6, 9.2)	3.51	>0.001
5	Non-dependence	54.3 (29.3)	51.6 (28.4)	2.6 (-0.9, 6.2)	1.45	0.148
6	Enjoy life	60.1 (24.2)	53.9 (23.0)	6.2 (3.3, 9.1)	4.22	>0.001
7	Meaningful life	67.5 (24.1)	63.6 (21.9)	3.9 (1.1, 6.7)	2.71	0.007
8	Absent guilt	79.2 (29.2)	79.0 (28.7)	0.2 (-3.4, 3.7)	0.10	0.925
9	Sure future	68.5 (30.0)	66.7 (26.0)	1.9 (-1.6, 5.3)	1.08	0.283
10	Sure living	68.8 (29.3)	68.8 (27.8)	0 (-3.5, 3.5)	-0.02	0.989
11	Concentration	59.3 (20.4)	55.2 (21.3)	4.0 (1.5, 6.6)	3.10	0.002
12	Perceived safety	63.9 (19.1)	59.4 (19.5)	4.5 (2.2, 6.9)	3.74	>0.001
13	Physical environment	68.9 (15.4)	63.9 (20.1)	5.0 (2.8, 7.2)	4.43	>0.001
14	Energy level	74.8 (22.6)	71.0 (24.0)	3.8 (1.0, 6.7)	2.62	0.009
15	Bodily appearance	71.8 (20.3)	66.7 (24.9)	5.1 (2.3, 7.8)	3.56	>0.001
16	Financial level	48.7 (26.5)	43.7 (24.6)	5.0 (1.8, 8.1)	3.12	0.002
17	Public acceptance	65.7 (25.2)	59.7 (24.9)	6.0 (3.0, 9.1)	3.85	>0.001
18	Information access	62.0 (19.3)	58.8 (19.9)	3.2 (0.8, 5.6)	2.64	0.008
19	Leisure activities	63.7 (23.0)	58.9 (24.0)	4.8 (1.9, 7.7)	3.27	0.001
20	Getting around	78.9 (78.2)	68.9 (20.1)	10.0 (3.0, 17.0)	2.81	0.005
21	Sleep satisfaction	75.5 (22.0)	74.9 (22.8)	0.6 (-2.1, 3.4)	0.45	0.652
22	Satisfied with ADL	77.5 (16.6)	73.3 (19.2)	4.2 (2.0, 6.4)	3.74	>0.001
23	Work capacity	78.3 (15.1)	70.3 (19.0)	8.0 (5.9, 10.1)	7.43	>0.001
24	Self-satisfaction	74.6 (19.3)	71.6 (21.7)	2.6 (0.1, 5.1)	2.02	0.043
25	Relationship	70.5 (21.2)	63.3 (25.3)	7.2 (4.3, 10.1)	4.92	>0.001
26	Sex life	62.3 (23.2)	56.7 (27.7)	5.7 (2.5, 8.8)	3.53	>0.001
27	Friend support	66.8 (26.4)	63.8 (27.8)	2.9 (-0.4, 6.3)	1.73	0.083
28	Residence	73.4 (21.0)	67.7 (25.6)	5.7 (2.8, 8.6)	3.89	>0.001
29	Health service	74.3 (23.5)	74.6 (25.6)	-0.4 (-3.4, 2.6)	-0.25	0.800
30	Transportation	62.9 (26.5)	53.2 (29.2)	9.8 (6.4, 13.2)	5.61	>0.001
31	Positive feeling	72.7 (21.9)	63.2 (26.3)	9.5 (6.5, 12.4)	6.26	>0.001

SD: standard deviation; MD: mean difference

**Figure 1:**
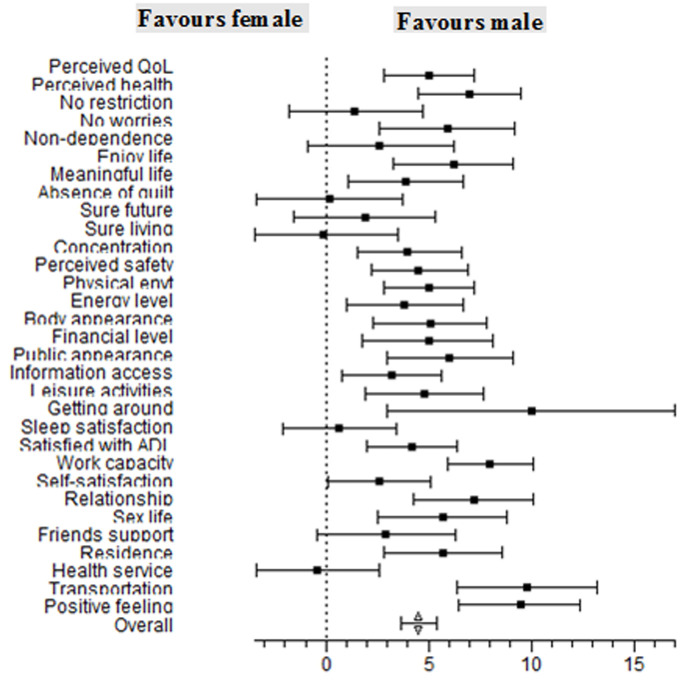
gender differences across items and entire WHOQoL HIV-BREF scale

The forest plot reveals the higher scores of male HIV patients over their female counterparts in 29 out of the 31 items of equal weights in the scale. The overall difference of the effect of gender on HRQoL between male and female HIV patients attending the ARV clinic was found to be 4.51% (95% CI of 3.63 to 5.39%) in favour of the male HIV patients. This difference was statistically significant with a p-value <0.001 (Figure 1). From Table 3, the percentage difference in the mean HRQoL scores of male and female across all domains in the HRQoL scale ranged from 1.48 (spiritual domain) to 6.2% (level of independence). The effect size statistics determined by the standardised mean differences showed a moderate effect of gender on the HRQoL of HIV patients along domains except for physical health (Cohen d = 0.17; 95% CI: 0.05, 0.30) and spiritual health (Cohen d = 0.07; 95% CI: -0.05, 0.20). The observed differences were all significant except for the spiritual domain (t = 1.19, df = 1022, p = 0.235).

**Table 3:** comparison of mean scores of HRQoL domains in male and female HIV patients

HRQoL Domain	Sex		Mean difference	Standardized mean difference (95% CI)	t-test statistics (df)	p- value
	Male - mean score (95% CI)	Female - mean score (95% CI)				
Physical health	76.06 (74.71, 77.39)	73.12 (71.44, 74.75)	2.95	0.17 (0.05,0.30)	2.76 (1022)	0.006
Psychological domain	67.60 (66.42, 68.86)	62.12 (60.57, 63.80)	5.48	0.34 (0.21,0.46)	5.34 (1022)	<0.001
Level of independence domain	72.23 (70.37, 74.29)	66.03 (64.76, 67.34)	6.20	0.31 (0.19,0.44)	5.00 (1022)	<0.001
Social relationship domain	66.31 (64.88, 67.69)	60.86 (59.05, 62.69)	5.45	0.31 (0.18,0.43)	4.93 (1022)	<0.001
Environmental health	64.71 (63.78, 65.68)	60.02 (58.60, 61.46)	4.69	0.34 (0.21,0.46)	5.38 (1022)	<0.001
Spiritual domain	71.00 (69.10, 72.85)	69.52 (67.78, 71.22)	1.48	0.07 (-0.05,0.20)	1.19 (1022)	0.235

CI: confidence interval; df: degree of freedom; statistical significance (p<0.05)

Table 4 presents data on patient-level predictors that could interfere with the gender determinant of HRQoL. The employment status and age of the patients were not significant predictors of HRQoL. The multivariate regression model that included all significant predictors of HRQoL from the bivariate analyses was significant at predicting a change in the scores of HRQoL of HIV patients [F (10, 1013) = 37.77, p<0.000]. While gender in a univariate analysis can explain 3.2%, this multivariate model including all significant predictors in the univariate models explains 27.2% of the variance of HRQoL scores of HIV patients attending UPTH with a Durbin-Watson statistic of 1.63 (Table 4). All variables in the multivariate model were significant predictors of HRQoL of HIV patients after controlling for their interacting, effects except the clinical state of the patient which was not significant (B = -0.7; 95%CI: -2.2, 0.8; p = 0.360).

**Table 4:** patients' background characteristics associated with HRQoL

Independent variable	Mean (SD)	Bivariate analysis		Multivariate analysis	
		B (95%CI)	p-value	B (95%CI)	p-value
**Gender**			<0.001		<0.001
Female	64.6 (14.0)	-		-	
Male	69.1 (10.9)	4.5 (3.0, 6.1)		3.5 (2.1, 4.9)	
**Marital status**					
Single	65.5 (14.9)	-	-	-	
Married	69.0 (10.2)	3.4 (1.8, 5.1)	<0.001	4.2 (2.8, 5.7)	<0.001
Separated/divorced	60.5 (14.1)	-5.0 (-10.1, 0.1)	0.053	-4.8 (-9.3, -0.4)	0.034
Widowed	62.9 (6.7)	-2.7 (-6.2, 0.9)	0.143	-3.2 (-6.3, -0.1)	0.044
**Level of education**					
No formal education	49.7 (28.1)	-	-	-	
Primary	63.5 (16.3)	13.8 (8.4, 19.1)	<0.001	7.1 (2.3, 12.0)	0.004
Secondary	66.8 (9.9)	17.0 (12.1,22.0)	<0.001	11.1 (6.6, 15.6)	<0.001
Tertiary	69.8 (12.5)	19.8 (14.8, 24.8)	<0.001	14.0 (9.4, 18.6)	<0.001
**Family type**			<0.001		<0.001
Monogamous	68.4 (11.0)	-		-	
Polygamous	61.8 (16.2)	-6.6 (-8.4, -4.8)		-4.9 (-6.6, -3.3)	
**Perceived heath status**			<0.001		<0.001
Not good	51.2 (23.0)	-		-	
At least good	68.4 (9.9)	17.2 (14.8, 19.7)		14.1 (11.6, 16.6)	
**HIV clinical status**			0.003		0.360
Symptomatic	65.3 (15.5)	-		-	
Asymptomatic	67.8 (10.6)	2.5 (0.9, 4.1)		-0.7 (-2.2, 0.8)	
**Employment status**			0.249		
Yes	66.1 (12.8)	-			
No	67.1 (12.7)	1.0 (-0.7, 2.7)			
**Age**			0.258		
≤ 40 years	66.6 (13.3)	-			
> 40 years	67.6 (11.3)	1.0 (-0.8, 2.8)			

## Discussion

This study found that male HIV patients fared significantly better than their female counterparts in about three-quarter of the items in the WHOQoL-HIV-BREF scale and overall scored 4.5% higher mean HRQoL which was statistically significant. There was similar finding along domains except for the spiritual domain. Other factors associated with significantly lower HRQoL were being single, lower level of education, polygamous family type and good self-rated health status.

The HRQoL is essential for determining the impact of chronic disease on a patient and often correlate with the overall outcome of a disease [6]. The WHOQoL-HIV-BREF scale used in this study was found to have satisfactory measurement properties which are critical to providing reliable and valid data [14]. The need to meet up with the financial cost of their daily needs was of utmost concern to male and female HIV patients in this study. Apart from the risk of suffering lower economic productivity due to HIV infection, PLWHA has increased daily needs and experience more hassles with healthcare [15]. Although waivers are available for the cost of drugs and specific investigations related to monitoring ART, patients still bear the brunt of other medical and non-medical cost as they seek healthcare. Such payments are mainly through out-of-pocket which often affect household finances and result in the inability of these households to meet other legitimate obligations [16].

Significant disparities in HRQoL were observed in about 75% of items in the WHOQoL-HIV-BREF scale. The significantly higher HRQoL among male patient is a consistent trend in the literature [7,17]. This forms part of the direct consequence of female subordinate position and the greater risk of experiencing deprivation to access to education, health services, independent income, property and legal rights [18]. Indeed, the lower HRQoL often observed among female HIV patients [17] may be attributable to the socio-economic climate in many developing countries, prevalent HIV-related stigma; persisting gender-related inequality in economic and social status; discriminatory access to healthcare and supportive services [4,7,19].

The finding of environment and social relationship domains being the lowest HRQoL scores was corroborated by earlier reports from South-West Nigeria [20] and Zhejiang province [21]. While significant gender-based disparities observed in all domains except the spiritual/religion/belief domain, previous studies have shown female having significantly lower scores in all seven domains of the WHO-HIV-BREF in a Northern Ethiopian study [22], five of the seven domains except social and spiritual in Ethiopia [7] and no significant difference reported in any of the four domains in an Indian study [23]. As a contrast to these findings, other studies from North-West Nigeria, South-West Nigeria and Delhi, India showed females having higher scores in most domains of the HRQoL scale even though these differences were not all statistically significant [20,24,25]. Besides the earlier mentioned female subordination and derivation, the domestic burden of home-keeping is traditionally performed by women in many societies and illnesses in women like other needs are taken less seriously and attract less family and social support than the male counterparts [7]. In more traditional societies like in Nigeria, women are often blamed for their health predicaments and sometimes assigned abusive tags such as “flirts” while men are exonerated even when they are involved in risky sexual behaviours [26].

Similar scores observed in both groups in the spiritual domain like in a previous study [7], indicates that males and females alike become more spiritual when confronted with problems considered to be beyond their capacity to handle. Unique values to individuals like spirituality and religiosity which aim at creating meaning and satisfaction in the usual circumstances people faced in life have been associated with a variety of positive health outcomes [27]. These coping strategies should be given serious consideration in planning public health interventions to improve the HRQoL of HIV patients. Female HIV patients do not only differ in HRQoL but also in other socio-demographic, economic and clinical characteristics. The interacting effects of socio-economic status and psychological factors in a model can obviate the influence of gender on the HRQoL of PLWHA. Whereas women occupy the lower end of the socio-economic and educational ladder [7], the association between low socio-economic and educational status and lower HRQoL has been established [20,21]. Although gender still had a strong association with HRQoL after including these variables in the same model, it is still pertinent to explore and remediate other identified modifiable factors associated with lower HRQoL among female HIV patients. Strategic interventions should aim at improving the education of women at least up to the secondary level. A female who attains such a high level of education are less likely to marry and get pregnant as adolescents and found to possess the right knowledge and skills for economic productivity [28].

Stigmatization and discrimination result from individual, family and societal reactions toward PLWHA. The finding that male and female HIV patients were least bordered about the public reaction to their HIV status, signal a positive turn of psycho-social reactions that have been a formidable challenge to prevention and control of the scourge in many traditional societies [29]. This curious observation shows that HIV patients get accustomed to their condition over time and no longer feel threatened by stigma and discrimination [30]. Such inherent coping ability may be a confounder factor in reported gender differences in HRQoL [31]. While family can provide significant stress and support for PLWHA, family and friends support as well as a positive psycho-social disposition by the patient can improve quality of life and produce desired outcomes in PLWHA [30,31]. As an issue of human right, the ´90-90-90 targets´ by the Joint United Nations Programme on HIV and AIDS and partners in 2014 aimed to diagnose 90% of all HIV positive people, provide ART for 90% of those diagnosed and achieve viral suppression for 90% of those on treatment [32]. The decreasing mortality and incidence of new infection following improve access to HAART had changed the nature of HIV disease from terminal to long-term. The disproportionately low resources available for provision of non-personal health needs and empowering vulnerable groups with tools for safe behaviours, leaves PLWHA with significantly lower HRQoL than the general population [21]. Among this group, the poorer wellbeing of female HIV patients calls for targeted strategies aimed at improving their HRQoL and reducing gender-based disparities among PLWHA.

While gender equality is an important target of the Sustainable Development Goal five [33], understanding the biological, social, cultural, religious, economic, and legal factors that predispose women to HIV infection [1,4,8], and how these factors impact on their HRQoL is a critical step in providing solutions to the problem. The dismal consequences of gender inequalities and low priority given to women´s rights which is depicted in the feminization of the HIV epidemic in this setting, provide a strong basis for implementing universal coverage for integrated sexual and reproductive health services that are designed to meet the needs and rights of women especially those living with HIV and AIDS.

Although gender depicts the social and cultural role of male and female and not just their biological identities, the significantly poorer HRQoL of female HIV patients may call for investigation into possible nexus between the cultural roles of women and their wellbeing. In addition to their vulnerability to HIV infection in this setting [4,18], there are indications that women face greater challenges with coping with their condition. A focus on gender mainstreaming and the implementation of family-centered interventions in the management of HIV may result in significant improvement in the HRQoL of female HIV patients. Policies and strategies in this regard should aim at bridging the wide social, cultural, education and income gaps between male and female. This can be achieved when government at the federal, states local levels are held accountable for implementing and enforcing extant provisions of the law that protects the rights of women. That most patients were bothered about their ability to meet up with their financial obligations underscores the low level of economic empowerment. Economic empowerment will entail securing adequate access to financial services such as savings, insurance, credits, and remittances for women. The reality that only 27% of women compared to 51% of men currently own personal account or use a mobile money service in Nigeria [28], provides further imperative for vigorously pursuing public and privately financed women economic empowerment initiatives.

Unemployment was reported to be higher among women in this study and generally in Nigeria; as such, targeted economic empowerment is needed to enhance their bargaining power, wellbeing, and ability to cope with the various challenges of family responsibility and care of themselves [28]. While commending the federal government for introducing the 'government enterprise and empowerment programme´ (GEEP) often called “TraderMoni”, it is pertinent to strengthen its implementation framework and accountability mechanism that will shield it against fraud and politicization [34]. This is required to reduce the current income and wealth inequality in the country. The influence of individual and societal culture on sickness and healing cannot be overemphasized achieving gender parity in HRQoL may be elusive if the current patriarchal culture and power dynamics that demand silence amid discrimination, abuses and economic constraints against women is reformed [19]. Legislations that would reverse the subordinate social status of women and their economic dependencies would assure equity in access to social support, empower women to resist coercion and be able to negotiate safer sex [19].

**Study limitations:** this study was limited by recall bias as often associated with a self-administered questionnaire. Although this study was conducted in only one of the two referral centres which has the largest pool of HIV patients on ARV, the generalisation of the findings to the state and national population of adult HIV patients on treatment remains guarded. Important clinical and laboratory markers such CD4+ count, duration of HIV infection and presence of co-infection which could influence the HRQoL [7,10,20,35] were not captured in this study. The data on earnings or wealth was not included in the analysis even though this is critical in a setting where access to healthcare is dependent on the ability to pay [16]. Finally, the cross-sectional nature of this study limits the application of causal inferences from the findings.

## Conclusion

Despite decreasing HIV mortality and incidence of new cases in recent times, the significant disparities in the HRQoL of male and female HIV patients calls for strengthening policies on gender mainstreaming in the management of HIV and broadening scope of HIV services to cover essential physical, psychological, social, environmental, and spiritual needs of the PLWHA. The development of appropriate indicators to monitor progress toward improving the HRQoL of HIV patients in this setting should form a part of the plan of action.

**Funding received for this study:** partial funding was provided for the dissemination of research finding by the Africa Centre of Excellence in Public Health and Toxicological Research (ACE-PUTOR), University of Port Harcourt.

### What is known about this topic

- HIV infection is still a significant contribution to morbidity, disability, and mortality globally;
- People living with HIV/AIDS (PLWHA) now live longer because of increasing access to highly active anti-retroviral therapies;
- The health-related quality of life (HRQOL) of PLWHA is now a critical concern.

### What this study adds

- There are significant differences in HRQOL between male and female PLWHA in Nigeria along items, domains, and entire WHO-HIV-BREF scale;
- Male HIV Patients showed significantly higher HRQOL than the female counterparts;
- Observed differences call for strengthening gender mainstreaming in the provision of HIV services in Nigeria.
